# New insights into discrepancies between self-reported and accelerometer-measured moderate to vigorous physical activity among women – the mPED trial

**DOI:** 10.1186/s12889-016-3348-7

**Published:** 2016-08-11

**Authors:** Yoshimi Fukuoka, William Haskell, Eric Vittinghoff

**Affiliations:** 1 Institute for Health & Aging / Department of Physiological Nursing, School of Nursing, University of California, San Francisco, 3333 California Street, Suite 340, San Francisco, 94118 USA; 2 Stanford Prevention Research Center, Stanford University, 1070 Arastradero Rd. Suite 100, Palo Alto, CA 94304 USA; 3 Department of Epidemiology & Biostatistics, University of California San Francisco, 550 16th Street, San Francisco, CA 94158 USA

**Keywords:** Accelerometer, Physical activity, Self-reported questionnaire, Moderate to vigorous physical activity, Women, Randomized controlled trial

## Abstract

**Background:**

The aims of this report were 1) to describe the duration of moderate to vigorous physical activity (MVPA) and the proportion of participants meeting the recommended criterion of at least 150 min of MVPA per week as measured by the 7 Day Physical Activity Recall Questionnaire (7D-PAR) and accelerometer among women who were enrolled in the mPED trial; 2) to assess the level of agreement of the two measures using a Bland-Altman plot; and 3) to describe the positive and negative predictive values (PPV and NPV, respectively) of meeting the guidelines by calculating the percentage of women meeting the physical activity recommendation by the 7D-PAR who also met this recommendation based on data from the accelerometer.

**Methods:**

Baseline data on duration of MVPA from the mPED trial were analyzed for 215 women. Among the women who met the recommended criterion by the 7D-PAR (self-report), we calculated the proportion of individuals who also met it by the accelerometer (objective measure). A Bland Altman Plot was used to assess concordance between the two measures.

**Results:**

The mean age was 52.4 (±11.2) years; 54.4 % were white; and 48.8 % were single or divorced. While median MVPA was 160 min/week by the 7D-PAR, it was only 24 min/week in the accelerometer. A total of 117 women met the 150-min criterion by the 7D-PAR. Of these, only 18 also met the criterion by the objective measure (PPV 15.4 %, 95 % CI 9.4–23.2 %). Among the 98 women who did not meet the criterion by the 7D-PAR, none met it by the accelerometer (NPV 100 %). A Bland Altman plot showed the mean difference of +145 min between the two measures with a 95 % limit of agreement at + 471 to −181 min.

**Conclusions:**

The large discrepancy between the self-reported and objective measures of MVPA meeting the 150-min criterion suggests that self-reported physical activity measures should be used with caution in intervention studies. While our data suggest that self-report could be used to identify a physically inactive sample, it would be likely to over-estimate the proportions of women who become active in one or both arms of trials of interventions promoting MVPA.

**Trial registration:**

ClinicalTrials.gov NCT01280812

## Background

Increasing physical activity is associated with reduction in chronic illnesses, such as hypertension and type 2-diabetes [1–3]. The 2008 Physical Activity Guidelines for Americans recommends U.S. adults to engage in a total 150 min of moderate-intensity aerobic activity (i.e., brisk walking) every week or 75 min of vigorous-intensity aerobic activity every week, to be done with at least 10 min bouts of activity [4]. The self-reported data from the national surveys suggested that approximately 50 % of all adults met the 2008 Physical Activity Guidelines [5]. In contrast, the accelerometer-based objectively measured data indicated that only a small proportion of the adults met the guidelines [5]. A large gap between self-reported and objectively measured physical activity levels exists at population levels [6].

Accurate measurements of physical activity are important to evaluate the efficacy or effectiveness of interventions designed to increase physical activity levels over time. Self-reported questionnaires are the most commonly used tools to assess changes in physical activity [5] because they are easy to complete within a short time period and can be administered at relatively low cost. However, a recent systematic review pointed out that self-report measures are susceptible to both overestimation and underestimation of true physical activity levels [6]. Recall bias, response bias, social desirability, and inability to understand levels of intensity are often considered to be the sources of the inaccuracy [7, 8]. Understanding the degree of discrepancy in physical activity levels between self-report and objective measures has significant public and scientific implications for designing physical activity intervention studies.

A recent systematic review comparing direct versus self-reported measures for assessing physical activity in adults reported several limitations of the current evidence [6]. First, the majority of the reviewed papers (148 out of 173 papers) only examined a correlation between the two measures, but did not report the level of agreement and systematic bias [6]. Second, inconsistency in the number of days measured, measurement time lag, and the unit of physical activity reported between the self-reported and objective measures made it difficult to make a direct comparison of these two measures. Lastly, a small sample size appeared to be an issue. Only 3.4 % and 1.2 % of the papers included a sample size between 100 and 200 and greater than 200 participants, respectively. Therefore, it is important to take into account all of these issues when designing a study to compare self-reported and objectively measured physical activity.

This **m**obile phone based **p**hysical activity **ed**ucation (mPED) study is a randomized controlled clinical trial (RCT) with a run-in procedure and is designed to evaluate the efficacy of a mobile app and accelerometer delivered physical activity intervention for physically inactive women [9, 10]. The baseline physical activity data measured by the 7-Day Physical Activity Recall Questionnaire (7D-PAR) (self-report) and accelerometer (objective measure) provide a unique opportunity to explore the level of agreement between the two measures by addressing all of the limitations described above. The aims of this paper are: 1) to describe the duration of moderate to vigorous physical activity (MVPA) and the proportion of participants meeting the recommended criterion of at least 150 min of MVPA per week as measured by the 7D-PAR and accelerometer among women who were enrolled in the mPED trial; 2) to assess the level of agreement of the two measures using a Bland-Altman plot; and 3) to describe the positive predictive value (PPV) of meeting the guidelines by calculating the percentage of women meeting the physical activity recommendation by the 7D-PAR who also met this recommendation based on data from the accelerometer. We believe that the findings of this study can assist in designing physical activity measures and interventions for physically inactive women in the near future.

## Methods

### Study design, sample, and recruitment

**Fig. 1:**
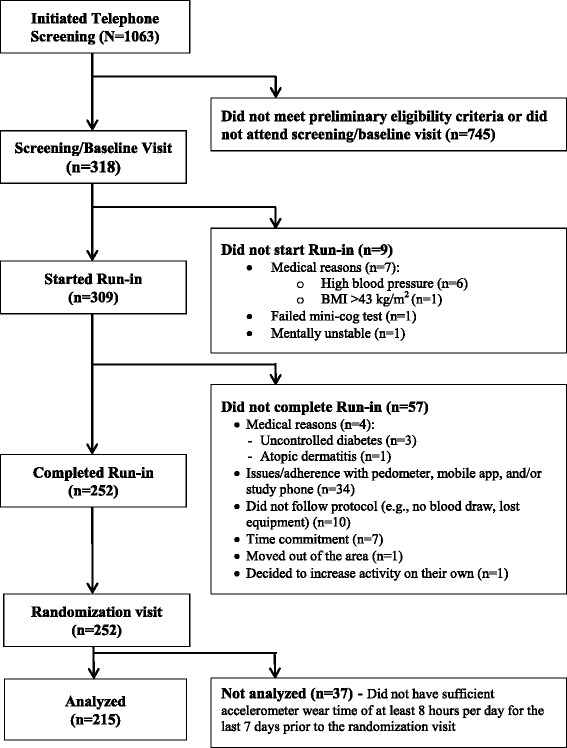
Study design and participant enrollment process

In this cross-sectional study, the baseline physical activity data of the mPED study were analyzed to compare MVPA measured over 7 days by the 7D-PAR and accelerometer. Figure 1 shows the study design and participant enrollment process. The study protocol was approved by the University of California, San Francisco Committee on Human Research (CHR) and the mPED Data and Safety Monitoring Board. The study protocol has been previously published [9, 10]. Physically inactive women were recruited from the San Francisco Bay Area between May 2011 and April 2014. With the aim of recruiting a diverse and representative sample, four broad types of subject recruitment strategies were used: 1) media advertising (e.g. newspaper, radio, Craigslist, and Facebook ads; email distribution lists; study, clinic, and clinicaltrials.gov websites); 2) posting fliers in the community (e.g., stores, bus stops, medical and dental clinics, community centers, university campuses, and churches), 3) random mailing of the study announcement to women age 25–65 who live in San Francisco, and 4) referral from friends, family members, health care providers, or others contacts.

Subjects were initially screened for inclusion and exclusion criteria by telephone and then assessed during an in-person visit. Inclusion criteria were: 1) physically inactive at work and/or during leisure time, based on the Stanford Brief Activity Survey [11]; 2) intent to be physically active; 3) female, age 25 – 69 years; 4) access to a home telephone or mobile phone; 5) speak and read English; 6) body mass index (BMI) of 18.5–43.0 kg/m^2^. Exclusion criteria were: 1) known medical conditions or physical problems that require special attention in an exercise program; 2) planning an international trip during the next four months (which could interfere with daily server uploads of mobile phone data); 3) pregnant/gave birth during the past six months; 4) severe hearing or speech problem; 5) history of eating disorder; 6) current substance abuse; 7) current participation in lifestyle modification programs or research studies that may confound study results; 8) history of bariatric surgery or plans for bariatric surgery in the next 12 months; or 9) no mild cognitive impairment as determined by the Mini-Cog test [12, 13]. In addition, women who did not complete the 7D-PAR questionnaire or did not have sufficient accelerometer wear time of at least 8 h per day for the last 7 days of the randomization visit were excluded from this study.

As seen in Fig. 1, 318 participants came in for the screening/baseline visit. Of these, 9 did not meet at least one of the inclusion criteria, 57 did not complete the run-in period (no 7D-PAR data), and 37 did not have sufficient accelerometer wear time of at least 8 h per day for the last 7 days prior to the randomization visit. The remaining 215 participants were analyzed in this report. All demographic data, except for age, were similar among the three groups. The mean age in the sample of 215 was significantly higher than the other two groups (*p* = 0.029).

### Study visits

#### Telephone screening

During the initial screening call, a trained study staff member screened potential participants for preliminary eligibility. Potential participants who met preliminary eligibility criteria were invited to attend a screening/baseline visit and received the study consent form, public transportation and parking information, directions to the research office, and a list of study requirements, which included a picture of the accelerometer that they would be asked to wear.

#### Screening/Baseline visit

In total, 318 women came in for a screening/baseline visit (Fig. 1). All participants provided written consent prior to study enrollment. Participants were first screened for mild cognitive impairment using the Mini-Cog test [12, 13]. Sociodemographics, medical and lifestyle history, Modified Self-Efficacy for Physical Activity Survey, Social Support and Exercise Survey, and Barriers to Being Active Quiz, Center for Epidemiological Studies Depression Scale (CES-D), and Television and Computer Usage questionaire developed by the intervesgtor were administered. A research staff conducted a physical exam. At the end this visit, eligible participants were issued a run-in app and an accelerometer and brief training was provided to insure participants could successfully use both devices.

#### Run-in period

The run-in period lasted approximately 3 weeks. A run-in mobile app was created specifically for this phase of the study which did not contain any content to encourage or support increasing physical activity. The participants were instructed to use this run-in mobile app at least twice a day (responding to a daily message and recording in a diary at night) every day during the run-in period. For example, the run-in app sent a daily message per day throughout the run-in period, and participants were instructed to respond to each message. A sample message would be: “are you wearing a pedometer right now ?” If the response was “no,” a reminder message to wear a pedometer all day was sent to the participant. In addition, participants were instructed to enter whether they wore the pedometer all day. If the answer was “yes,” they were asked for an estimate of their daily step count into the app every night of the run-in period. The app could be installed on a participant’s personal phone if they had a compatible smartphone. Alternatively, participants were provided with a phone for the purpose of the study.

#### Randomization visit

During the randomization visit, trained research staff administered the 7D-PAR to assess the last 7 days of physical activity. Participants were asked to return the accelerometer to research staff, and research staff downloaded the data onto the study computer to check their wear time and average numbers of steps they had taken daily during the run-in period.

### Measures

All questionnaire data below were collected on machine-readable data forms based on the Cardiff Tele-form software system. Completed teleforms were faxed to the San Francisco Coordinating Center where a server using optical character recognition (OCR) technology verified and stored the data in the study database on a secure Microsoft SQL server. A standardized procedure was used to convert the SQL data to SAS data [9, 10].

#### Sociodemographic, lifestyle, and anthropometric measures

Sociodemographic and lifestyle information were collected during the telephone screening or screening/baseline visit. Before anthropometric measures, all participants were asked to change to a hospital gown and remove their shoes prior to measurements. Height, weight in kilograms, and waist circumference were measured and body mass index (BMI) calculated. BMI was calculated based on height and weight in kilograms that were measured during the screening/baseline visit.

#### Modified self-efficacy for physical activity scale

A 6-item modified version [14] of the original 5-item Self-Efficacy for Physical Activity Scale [15] was used to assess confidence in one’s ability to exercise, an important determinant of the stages of change for exercise behavior. Total scores can range from 6 to 30, with higher scores indicating greater self-efficacy for physical activity.

#### Social support for exercise survey

It consists of 13 items assessing the level of perceived support from family and friends for behavior changes related to exercise [16]. Each item is scored separately for family and friends, and scores can range from 13 to 65 with higher scores indicating greater support.

#### Barriers to being active quiz

It consists of 21 items assessing 7 types (subscales) of barriers to physical activity: lack of time, lack of social influence, lack of energy, lack of willpower, fear of injury lack of skill, and lack of resources [17]. Each subscale can range from 0 to 9 and total scores can range from 0 to 63, with higher scores indicating more barriers to physical activity.

#### Center for Epidemiological Studies Depression Scale (CES-D)

It is a 20-item questionnaire widely used for assessing symptoms of depression [18]. Scores can range from 0 to 60, with higher scores indicating more depressive symptoms.

#### Seven- Days Physical Activity Recall (7D-PAR) (Self-reported measure)

The 7D-PAR is a semi-structured interview that estimates an individual’s time spent in moderate, hard, and very hard physical activity, and strength, and flexibility activities for the 7 days prior to the interview [19]. A trained research staff used the 7-day worksheet to assess at least 10 min of moderate, hard, and very hard physical activity each day for the 7 days. To help participants to understand their intensity of physical activity, the following examples of activity and intensity were provided: Moderate activities are similar to brisk walking as if you are in a hurry to get somewhere. Moderate activities also include household chores like sweeping, mopping, and vacuuming. Very hard activities included traditional aerobic activities like running. Hard activities are defined as activity requiring more effort than moderate but not as much as very hard activities. Specific daily activities, such as breakfast, lunch and dinner, were investigated to aid subjects’ memory of the activity’s intensity and duration. A weekly total-minute of moderate or vigorous intensity activity was calculated by summing up all qualified minutes during the past 7 days. The 7D-PAR manual was used to standardize the interview process, research staff, and increase agreement among research staff.

#### Accelerometer (Objective measure)

A triaxial accelerometer (HJA-350IT, Active style Pro, Omron Healthcare Co., Ltd.) was used to assess objectively measured physical activity [20, 21]. This accelerometer has been validated before, and a detailed description was published previously [20, 21]. In short, its dimensions are 74x46x34 mm (width/height/depth) including the clip, and it weighs 60 grams (2.1 oz.). Anteroposterior (x-axis), mediolateral (y-axis), and vertical (z-axis) accelerations were gathered from the triaxial accelerometer during each activity at a sampling rate of 32 Hz. The acceleration data are expressed relative to g (1 g = 9.81 m/s2). The maximum scaling of the acceleration data was ± 6 g (resolution 0.003 g) with a 12-bit analog-to-digital converter [20]. This accelerometer was programed to collect physical activity intensity (metabolic equivalent values (METs)) every 10 s and, the mean intensity value of a 1-min epoch was calculated as the average value of six 10-s epochs. METs determined by this accelerometer are closely correlated with METs calculated using energy expenditure measured by indirect calorimetry [20, 21].

The software program provided by the manufacturer (HMS-HJA-IC01J; Omron Healthcare Co., Ltd.) was downloaded to a study personal computer in the research office. A trained research staff entered the participant’s study ID, weight, height, age, and gender, into the accelerometer. The accelerometer was then set to display only date and time at the screening/baseline visit. To avoid providing any feedback and to collect the clean baseline activity data, neither the step counts nor metabolic equivalent values (METs) were displayed. At the screening/baseline visit, the following instructions were also provided to participants: 1) placing the accelerometer on the waist in the middle of right or left thigh of their dominant leg; 2) wearing the accelerometer from the time they got up in the morning until they went to bed at night every day except when showering, bathing, swimming, or sleeping at night; and 3) engaging in their regular daily activity, but not increasing this activity during the run-in period. The accelerometer’s data was automatically reset at midnight. Activity data were stored minute-by-minute for the entire duration of the run-in period, and at the end of the run-in period (randomization visit). A trained research staff downloaded the stored data to a study personnel computer with the software program described above.

### Data treatment and statistical analysis

In this analysis, only recorded accelerometer data during the 7 consecutive days prior to the randomization visit were used to make a direct comparison with the 7D-PAR. In order for accelerometer data to be valid, all 7 days of accelerometer activity needed to indicate at least 8 h/day of recorded wearing time for the device. Moderate or vigorous intensity activity was defined as between ≥ 3 to < 6 or ≥ 6 metabolic equivalents (METs), respectively using the Compendium of Physical Activity [22, 23]. To closely match with the 2008 Physical Activity Guidelines for Americans, total weekly minutes of MVPA were estimated as physical activity ≥ 3 METs lasting at least 10 min in duration. Since the 7D-PAR does not include bouts of MVPA of less than 10 min in duration, counts from the accelerometer needed to continuously remain above the 3 MET level for 10 min in order for it to be counted as “meeting guidelines”. Furthermore, the following additional analyses were conducted. First, because some of the previously published studies allowed an interruption of one or two minutes during the 10 min bout of MVPA [24, 25], we also estimated total weekly minutes of MVPA with this rule. Second, total weekly minutes of MVPA (≥3 METs) in bouts lasting at least 1, 3, 5, and 7 min in duration without allowing any interruption were separately calculated.

Proportions of women meeting the guideline of at least 150 min of MVPA per week for both self-report and accelerometer measures of MVPA were calculated. In addition, the positive predictive value (PPV) of meeting the guideline by self-report was calculated as the percentage of women meeting the physical activity recommendation by the 7D-PAR who also met it based on data from the accelerometer. Similarly, the negative predictive value (NPV) was calculated as the percentage of women not meeting the recommendations by self-report who also did not meet it by the accelerometer. The Bland and Altman method [26] was used to provide an indication of the systematic and random error between the 7D-PAR and accelerometer as measures of weekly minutes of MVPA, and 95 % limits of agreement were used for describing the total error between the two measures. P values less than 0.05 were considered statistically significant. All statistical analyses were performed in SPSS 22 or Stata 14.0.

## Results

### Baseline characteristics

Table 1 shows the baseline sample characteristics and physical activity data of 215 women who met the inclusion criteria with 7 days of valid accelerometer data with at least 8-h minimum wear time per day and having completed the 7D-PAR. Overall, the mean age was 52.4 (±11.2) years old, 54.4 % were white, 48.8 % were single or divorced, and 73.0 % were well educated, reporting college or graduate level educations. In addition, 49.3 % had used a step counter (pedometer) and 57.2 % had participated in diet/weight loss plan prior to the study enrollment. The majority of the sample (80.5 %) drove a car at least once per week. The majority of the sample did not engage physical activities (e.g. cycling and swimming) that were not generally captured by an accelerometer.

**Table 1:** Baseline sociodemographic characteristics (N = 215)

Sociodemographics	Mean (±SD) or % (n)
Mean Age year	52.4 (±11.2)
Education : High School/some college	27.0 (58)
College /Graduate school	73.0 (157)
Race : White	54.4 (117)
Non-white	45.6 (98)
Marital status : Married/co-habitating	51.2 (110)
Paid job : Paid full/part-time	72.6 (156)
Homemaker/retried/disabled	27.4 (59)
Mean Body Mass Index kg/m^2^	29.3 (±6.1)
Total Center for Epidemiologic Studies Depression Scale score^a^	9.7 (±8.1)
Previous a step counter (pedometer) usage in the past	49.3 (106)
Drives a car at least once a week	80.5(173)
Have a dog	18.6 (40)
Participated in diet plan	57.2 (123)
Have a gym membership	29.3 (63)
Types of physical activities that the accelerometer cannot capture in the past month	
Cycling	6.0 (13)
Rollerblading/skating	0 (0)
Swimming	2.8 (6)
Weight lifting	1.4 (3)

^a^Possible Scores can range from 0 to 60, with higher scores indicating more depressive symptoms

### Duration of the 7D-PAR (Self-Reported) and objectively-measured MVPA minutes per week

Based on self-report by the 7D-PAR, which ascertains MVPA in bouts of at least 10 min duration, the 215 women reported an average of 197 (SD ±175; median 160) minutes (Table 2). When applying the same 10-min minimum so as not to allow any interruption in the accelerometer data, the average weekly MVPA was 38 (SD ± 62; median 14) minutes. Almost all (99 %) of the MVPA was of moderate intensity. When the 10 min duration for allowing for a 1 or 2 min interruption was applied, the average weekly MVPA was 48 (SD ± 61; median 24) minutes. In contrast, when we included any bout of at least 1, 3, 5, and 7 min without allowing for any interruption, the accelerometer recorded a mean of 300 (SD ±147; median 285), a mean of 138 (SD ±104; median 120), a mean of 85 (SD ±87; median 56) and a mean of 59 (SD ±75; median 33) minutes of MVPA, respectively (Table 2).

**Table 2:** Baseline 7D-PAR (self-reported) and accelerometer (objectively measured) physical activity (N = 215)

Physical activity measures	Median /mean (±SD) or % (n)
7D-PAR (self-report)	
Weekly total minutes of MVPA with 10 min criteria	160 /197 (±175) or
Last week’s physical activity level, compared to the past 3 months	
Same	61.8 (133)
Less	24.2 (52)
More	14.0 (30)
Meeting a 150 min/week of MVPA recommendation with 10 min criteria	54.5 (117)
Accelerometer (objective measure)	
Weekly total minutes of MVPA by accelerometer with the 10 min criteria without allowing for any interruption	14/38 (±62)
Weekly total minutes of MVPA by accelerometer with the 10 min criteria allowing for a 1 or 2 min interruption	24/48 (±61)
Weekly total minutes of MVPA by accelerometer with the 7 min criteria	33/59 (±75)
Weekly total minutes of MVPA by accelerometer with the 5 min criteria	56/85 (±87)
Weekly total minutes of MVPA by accelerometer with the 3 min criteria	120/138 (±104)
Weekly total minutes of MVPA by accelerometer with the 1 min criteria	285/300 (±147)
Meeting a 150 min/week of MVPA recommendation by accelerometer	
10 min criteria not allowing any interruption	4.7 (10)
10 min criteria allowing for a 1 or 2-min interruption	8.4 (18)
7 min criteria not allowing any interruption	8.4 (18)
5 min criteria not allowing any interruption	17.2 (37)
3 min criteria not allowing any interruption	35.8 (77)
1 min criteria	87.0 (187)
Self-reported physical activity questionnaires	Mean (±SD)
Total self-efficacy for physical activity score	19.0 (±4.6)
Social support for physical activity : Total family score	31.2 (±9.4)
: Total friends score	31.6 (±8.4)
Barriers to being active 1. Lack of time	4.0 (±2.7)
(subscale scores) 2. Influence from others	3.5 (±2.1)
3. Lack of energy	3.9 (±2.6)
4. Lack of willpower	6.7 (±2.2)
5. Fear of injury	1.26 (±1.7)
6. Lack of skill	2.0 (±2.1)
7. Lack of resources	2.4 (±2.1)
Total television and computer usage (hours/week)	27.3 (±17.9)

Using the 10-min minimum for both measures without allowing for any interruption, 54.4 % (*n* = 117) of women reported at least 150 min of MVPA by self-report, but only 4.7 % (*n* = 10) met this goal of 150 min according to the accelerometer data without any interruption. Even when applying the 10-min duration by allowing for a 1 to 2 min interruption in the accelerometer data, only 8.4 % (*n* = 18) met the guidelines. If the 1-min minimum was used for the objective measure (accelerometer), 87 % of women respectively met at least 150 min of MVPA.

### Positive and negative predictive values of self-report of at least 150 minutes of MVPA

Overall, 117 out of 215 women reported at least the recommended weekly 150 min of MVPA on the 7D-PAR, and the remaining 98 women did not meet with this recommendation report. As seen Table 3, among these 117 women, only 18 also met the recommendation by accelerometer for positive predictive value (PPV) of 18/117 = 15.4 % (95 % CI 9.4–23.2 %). In contrast, negative predictive value NPV was 100 % (98/98).

**Table 3:** Classification of subjects into four groups based on accelerometer and the 7 Day-Physical Activity Recall (7D-PAR) (N= 215)

		Accelerometer measured physical activity		
		≥150 min of activity per week^a^	<150 min of activity per week^a^	Predictive value
7D-PAR	≥150 min of activity per week	Both 7D-PAR and accelerometer group 8.4 % (*n* = 18)	7D-PAR only group 46.0 % (*n* = 99)	Positive predictive value 18/117 = 15.4 %
	<150 min of activity per week	Accelerometer only group 0 % (*n* = 0)	Neither group 45.6 % (*n* = 98)	Negative predictive value 98/98 = 100 %

^a^ Accumulation of at least 10 min duration (allowing for 1 to 2 min interruption) of moderate to vigorous physical activity (MVPA)

### Concordance of duration of moderate to vigorous physical activity (MVPA) between the 7D-PAR and accelerometer

**Fig. 2:**
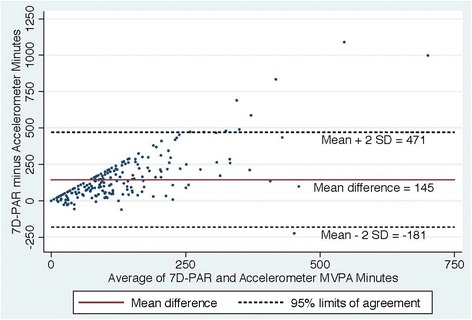
Bland Altman Plot of the differences in weekly duration of moderate to vigorous physical activity (MVPA**)** between the 7D-PAR and the accelerometer with the 10 min criteria allowing for a 1 or 2 min interruption

**Fig. 3:**
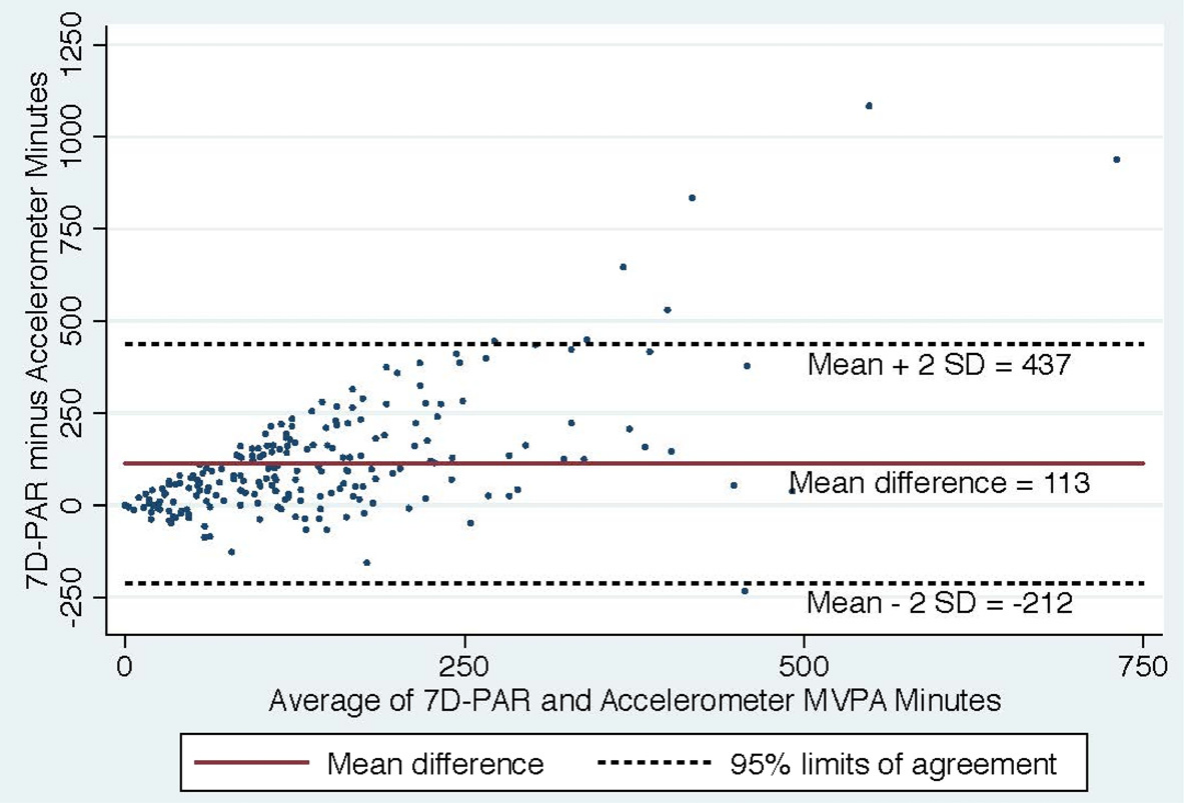
Bland Altman Plot of the differences in weekly duration of moderate to vigorous physical activity (MVPA**)** between the 7D-PAR and the accelerometer with the 5 min criteria

**Fig. 4:**
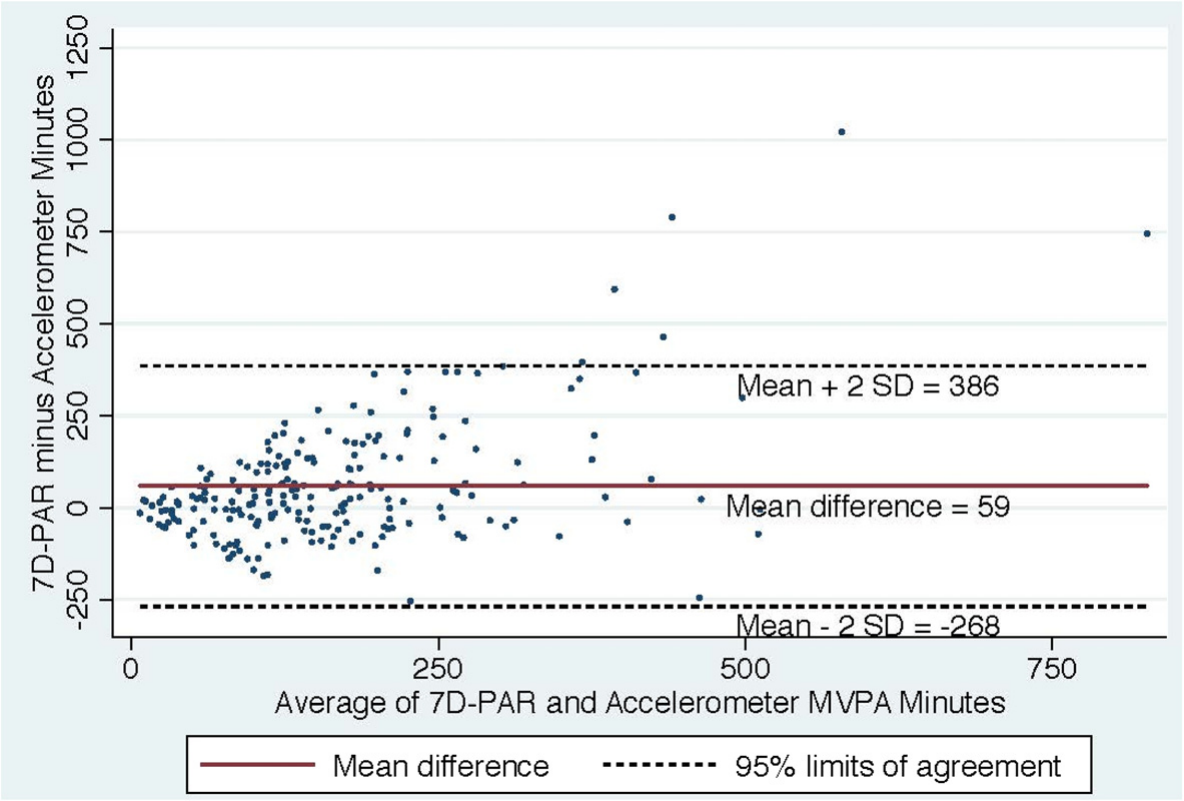
Bland Altman Plot of the differences in weekly duration of moderate to vigorous physical activity (MVPA**)** between the 7D-PAR and the accelerometer with the 3 min criteria

**Fig. 5:**
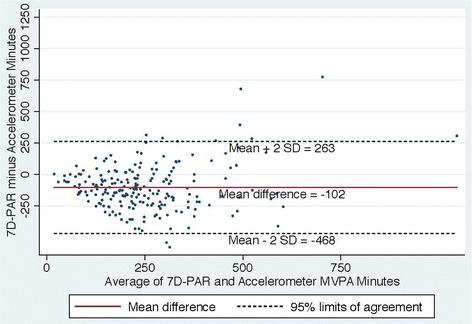
Bland Altman Plot of the differences in weekly duration of moderate to vigorous physical activity (MVPA**)** between the 7D-PAR and the accelerometer with the 1 min criteria

Figures 2, 3, 4 and 5 show Bland Altman plots of the agreement of the weekly duration of at least 10 min (allowing for a 1 or 2 min break), 5, 3 and 1 min in bout of MVPA between 7D-PAR and accelerometer during the run-in period. When the 10-min MVPA criteria allowing for a 1 to 2 min interruption was used, the agreement of the 2 measurements was extremely low (Fig. 2). The mean difference and 95 % limits of agreement were +145min with a 95 % limit of agreement at +471 to −181 min. Differences between the 7D-PAR and accelerometer scores increased as the average minutes of MVPA by the two measures increased. In contrast, with the 3-min MVPA criteria, the agreement of the 2 measurements became moderate (Fig. 4). The mean difference and 95 % limits of agreement were +59 min with a 95 % limit of agreement at + 386 to −268 min.

## Discussion

This study investigated the agreement between self-report and objective measures of MVPA among 215 women who completed the screening/baseline and randomization visits of the mPED trial. We found large differences in the median weekly total MVPA between the self-reported and objective measures when a 10-min bout of MVPA criteria with was applied. The agreement of the weekly duration of at least 10 min in bout of MVPA between 7D-PAR and accelerometer is poor despite allowing for a 1 or 2-min interruption in accelerometer data. Only 8.4 % of women who met the recommended criterion of 150 min of MVPA per week according to the 7D-PAR also met this criterion by the objective accelerometer-based measure. In contrast, all of the women who reported fewer than 150 min on the 7D-PAR had concordant qualitative accelerometer results. According to a recent systematic review of the comparison of direct versus self-report measures for assessing physical activity, self-report measures of physical activity were generally higher than those measured by accelerometers, in particular female adults [6]. A reported mean percent difference was 138 %, ranging from 100 % to 4024 % in the female adult only samples.

When at least 10 min in duration of MVPA allowing for a 1 or 2 min interruption was applied, the differences in weekly total minutes of MVPA reported on the 7D-APR and presented on the accelerometers increased, with an increase in the average weekly minutes of MVPA of the two measures (Fig. 2). The 7D-PAR assesses at least 10-min in duration and frequency spent in MVPA using each subject’s relative intensity, while the accelerometer uses absolute intensity across all subjects. This may partially explain the large average difference (+145 min with a 95 % limit of agreement at + 471 to −181 min) between the two measures, since the original aim of the RCT was to enroll physically inactive women who tended to have lower aerobic capacity. Interestingly, when at least a 3-min bout of MVPA criteria was used in the accelerometer data, the average difference between the two measures was the smallest (+59 min with a 95 % limit of agreement at + 386 to −268 min). The trend seen in Fig. 2 disappeared and the variability became more consistent across the graph in Fig. 4.

A clear strength of the accelerometer is that it can capture minute-by-minute activity. In contrast, most self-report measures, including the 7D-PAR, cannot ascertain MVPA with such fine resolution, and are also subject to reporting bias [27]. For these reasons, the accelerometer is becoming widely accepted as a gold standard measure of free-living physical activity. However, a disadvantage of the accelerometer measure is that no consensus method for summarizing the data has been established [28]. For example, in some of studies, an interruption of one or two minutes during the 10 min bout of MVPA has been allowed, since in developed countries intentional walking or running may be interrupted by traffic signals or safety concerns [24]. In addition, the minimum number of days required to calculate weekly average MVPA has varied across studies. In our study, only participants with validated data on all 7 days were included. In addition, we reported both data allowing and not allowing an interruption of 1 or 2 min during the 10-min bout of MVPA. We noted that more women would have met the 150-min criterion based on the accelerometer data if shorter bouts of MVPA had been allowed.

Troiano and colleagues first examined the 2003–2004 National Health and Nutrition Examination Survey (NHANES) which examined accelerometer measured physical activity data in a nationally representative U.S. sample [5]. They reported that only 2.3 % to 3.2 % female adults met the adherence of the recommendation of 150 min of MVPA per week by accelerometer, while approximately half of the sample met the recommendation by the self-reported measure. Although our sample differed substantially from the nationally representative NHANES sample, the findings of our study were similar: approximately half of our study sample met the recommendation by self-report, but only 8.4 % met it by accelerometer. As we discussed before, this large difference may be explained by over-estimation of the duration and/or intensity of physical activity in self-reports, with light intensity activities reported as moderate intensity, and/or bouts of less than 10 min included in the subjective totals. And while the accelerometer cannot capture activities such as swimming, bicycling, or weight-lifting, potentially resulting in underestimation of MVPA, these activities are relatively uncommon [5]. As seen in Table 1, the results of our baseline data confirmed this assumption in this sample. Furthermore, the sample NPV of 100 % indicates that if individuals did not meet with the recommendation by the self-reported measures, they were very unlikely to meet the recommendation by the accelerometer. In contrast, the low PPV suggests the need for caution in interpreting self-report in meeting the 150-min recommendation. Overall, the findings of this report highlight on how self-report might function as a proxy for accelerometer measurements for meeting physical activity recommendations.

### Strengths and limitations of the study

The strength of this study is that we were able to use the 7D-PAR and accelerometer measured physical activity data over the same time period, and to evaluate the objective measure to be consistent with self-report with a relatively large sample size. Another strength is that participants were not able to view their steps taken and intensity of physical activity during the run-in period. The Omron Active Style Pro HJA-350IT with triaxial accelerometer had a program option to select what types of information to be displayed. We believe that this blind function during the run-in period helped prevent participants from modifying their MVPA based on real-time feedback. Despite these strengths, some limitations need to be taken into account. First, the findings of this study might not be generalizable to men or children, nor to women who are unwilling to participate in an exercise trial like mPED. Second, the accelerometer used in the mPED trial was not able to capture activities such as swimming, bicycling, and weight lifting, in contrast to the 7D-PAR. However, our baseline data suggest that the prevalence of these activities in this sample is relatively low.

## Conclusion

The large discrepancy between the self-reported and objective measures of MVPA meeting the 150-min criterion suggests that self-reported physical activity measures should be used with caution in intervention studies. In particular, self-report of at least 150 min of MVPA had a PPV of only 15.4 %, although its NPV was 100 %. Thus while our data suggest that self-report could be used to identify a physically inactive sample, it could grossly over-estimate the proportions of women who become active in one or both arms of trials of interventions designed to increase MVPA.

## Abbreviations

MVPA, Moderate to intensive intensity physical activity; BMI, body mass index; CES-D, Center for epidemiological studies depression scale; kg, kilogram; m2, meter squared; mPED, Mobile phone based physical activity education; OR, odds ratio; PA, physical activity; RCT, randomized controlled trial; S/B, screening baseline; SD, standard deviation; SPSS, Statistical Package for Social Science; UCSF, University of California, San Francisco; US, United States; (7D-PAR), 7 Day Physical Activity Recall Questionnaire
